# Evolution of Thin-Film Wrinkle Patterns on a Soft Substrate: Direct Simulations and the Effects of the Deformation History

**DOI:** 10.3390/nano12193505

**Published:** 2022-10-07

**Authors:** Siavash Nikravesh, Yu-Lin Shen

**Affiliations:** Department of Mechanical Engineering, University of New Mexico, Albuquerque, NM 87131, USA

**Keywords:** thin film, wrinkle, deformation, surface instability, finite element analysis

## Abstract

Surface wrinkling instability in thin films attached to a compliant substrate is a well-recognized form of deformation under mechanical loading. The influence of the loading history on the formation of instability patterns has not been studied. In this work, the effects of the deformation history involving different loading sequences were investigated via comprehensive large-scale finite element simulations. We employed a recently developed embedded imperfection technique which is capable of direct numerical predictions of the surface instability patterns and eliminates the need for re-defining the imperfection after each analysis step. Attention was devoted to both uniaxial compression and biaxial compression. We show that, after the formation of wrinkles, the surface patterns could still be eliminated upon complete unloading of the elastic film–substrate structure. The loading path, however, played an important role in the temporal development of wrinkle configurations. With the same final biaxial state, different deformation histories could lead to different surface patterns. The finding brings about possibilities for creating variants of wrinkle morphologies controlled by the actual deformation path. This study also offers a mechanistic rationale for prior experimental observations.

## 1. Introduction

The formation of wrinkles (periodic buckles) on a thin film attached to a compliant substrate has received considerable attention in recent years. This form of surface instability is widely observed in nature and now increasingly encountered in flexible electronics and other functional devices [1,2,3,4,5,6]. Thin-film buckling can be triggered by the direct application of an in-plane compressive strain. It may also occur in response to applied tension, where a mismatch in lateral contraction between the thin film and a thick substrate forces the films into compression [7]. Wrinkling instability relaxes the compressive strain in the film layer, thus reducing the elastic strain energy. While the formation of wrinkles is traditionally viewed as an undesirable feature, it is increasingly exploited to enhance the mechanical and/or functional performances of energy and biomedical devices [8,9,10,11,12,13].

Theoretical solutions for the critical stress of wrinkle initiation, along with the wavelength and amplitude of the waveform, are available for the simplest forms of wrinkles [14,15,16,17,18]. The prediction of complex surface patterns resulting from the various loading, geometrical, and material conditions, however, will rely on computational means. Most numerical studies utilized the multi-step approach, with a pre-buckling step followed by a post-buckling analysis which essentially builds a waveform into the model [19,20,21,22,23,24,25]. The implementation can be laborious and lacks a true predictive capability. Other numerical techniques based on an applied fictitious/dummy load have been proposed [24,26,27,28], but multi-step analyses were still involved. Some of the recent studies followed similar approaches but focused more on wrinkling on curved composites [29,30]. Dynamic surface-instability simulations have also been conducted [31,32]; however, the verification and validation of the numerical outcomes are challenging, taking into account the lack of reliable experimental results or closed-form solutions to loading-rate-dependent deformation instability problems. Recently, a peridynamics analysis of wrinkling instabilities has also been reported [33,34], but the validity of non-local theories (such as peridynamics) in reproducing the actual physical phenomenon is still uncertain. Moreover, defining some of the free parameters such as the “horizon size” is also cumbersome.

The analytical/numerical studies of surface instability were mostly performed under the consideration of an instantaneous and non-sequential in-plane uniaxial or biaxial loading. The potential effects of different loading sequences on instability patterns, though leading to an identical final state of strains, have not been investigated. It is worthy of mention that the possibility of such effects on wrinkling configurations have been implied in some earlier experimental studies [15,35].

We recently developed a practical finite element modelling approach to simulate wrinkle formation using the embedded imperfection technique [36,37]. The temporal wrinkling evolution can be captured in a straightforward manner, and the methodology has been employed to study complex surface patterns in the three-dimensional (3D) setting [38,39]. The present work extends our previous 3D analysis to cover the effects of the deformation history. Using the model system of a polymeric thin film on an elastomeric substrate under in-plane uniaxial and equi-biaxial compressions, we first studied the evolution of the surface instability patterns and how they might gradually disappear when the applied compression was reverted back to zero. Note that our modelling approach allows such process to be carried out in one simulation run. In the case of biaxial loading, we further investigated the effect of the deformation history by comparing the surface patterns resulting from simultaneous loading and sequential loading along the two axes. Our results revealed the sensitivity of the wrinkling patterns to the loading sequence and identified new forms of surface pattern evolution affected by the deformation history.

## 2. Numerical Model Description

**Figure 1:**
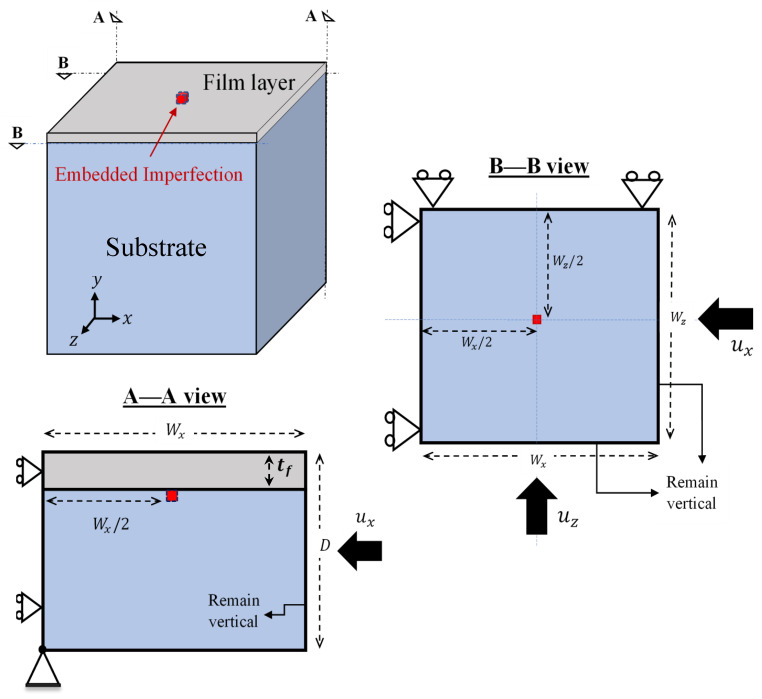
Schematics of the problem geometry, boundary conditions, and directions of the applied displacement [39].

The model consisted of a thin film bonded to a compliant substrate with an embedded imperfection. The film surface was square-shaped, and the substrate was sufficiently thick in comparison with the film. Figure 1 shows the problem geometry, boundary conditions, and directions of the applied displacement employed in the finite element analysis. All materials were chosen as linear elastic isotropic solids in the present study. The initial thickness of the film layer, *t_f_*, was 0.1 μm. One of the common polymeric film materials used in organic optoelectronics and photovoltaics, namely, P3HT:PCBM [8], was considered. The Young’s modulus of the thin film was *E_f_* = 7300 MPa [8], and the Poisson’s ratio was *ν_f_* = 0.35. The elastomeric substrate layer was PDMS, with Young’s modulus *E_s_* = 2.97 MPa [40] and Poisson’s ratio *ν_s_* = 0.495 (set slightly smaller than 0.5 to mitigate potential convergence issues). As schematically shown in Figure 1, the problem domain contained a single embedded imperfection. It was a regular finite element at the exact center of the top layer elements in the substrate, immediately below the film–substrate interface. The embedded imperfection, while underneath the interface in the substrate domain, was assigned the material properties of the film layer. This approach has been systematically tested and proven to be a reliable way to trigger deformation instabilities in our earlier studies [38,39,41].

Numerical simulations were performed using the finite element software ABAQUS (Version 2017, Dassault Systems Simulia Corp., Johnston, RI, USA). Throughout the model, 20-noded brick elements were used, with a uniform element distribution in the film layer (four layers of elements along the film thickness). An adapted element distribution existed in the substrate, with the element size increasing gradually from top to bottom. The in-plane mesh density of 10 elements per analytical sinusoidal wavelength was chosen to ensure mesh-convergent numerical solutions [38]. In addition to the mesh considerations, the displacement increment in the loading step was kept sufficiently small to avoid the potential increment dependency of the solutions [37]. The analyses were conducted via high-performance computing using the Message Passing Interface (MPI) parallelization technique. Preliminary calculations were carried out to ensure that the numerical results were independent of the parallel analysis procedures. Mesh convergence and model verification were also performed [38,39].

As shown in Figure 1, the roller boundary condition was imposed on the faces *z* = 0 and *x* = 0, and a corner point at the bottom of the substrate was fully fixed. The top face of the film and the bottom face of the substrate were kept traction-free. In addition, the faces *z* = *W_z_* and *x* = *W_x_* were constrained to remain vertical during the deformation. Therefore, the problem domain represented a unit cell of a large periodic structure [42]. The problem domain size was scaled by the value of the theoretical critical wavelength of the sinusoidal mode [14,16],

(1) $$\lambda_{cr}=2\pi t_{f}{\left[\frac{\left(1-\nu_{s}^{2}\right)E_{f}}{3\left(1-\nu_{f}^{2}\right)E_{s}}\right]}^{1/3}$$

The domain dimensions were $W_{x}=W_{z}=10\lambda_{cr}\;$(=55.92 µm) with the overall depth of *D* = $5\lambda_{cr}$. The dimensions of $W_{x}=W_{z}=10\lambda_{cr}$ were rationally chosen so that $10\lambda_{cr}≅7{\left(\lambda_{cr}\right)}_{Cb}$, where ${\left(\lambda_{cr}\right)}_{Cb}=\sqrt{2}\lambda_{cr}$ is the analytical wavelength of the square checkerboard instability mode under equi-biaxial compression [16]. Thus, the model size was well-suited to simulate various surface patterns in conformity with available analytical solutions. Displacement-controlled uniaxial and equi-biaxial compressions were considered. For uniaxial compression, a uniform displacement was applied in the *x*-direction only; for equi-biaxial compression, uniform displacements with equal magnitudes were applied concurrently in both the *x*- and the *z*-directions. The biaxiality ratio, BR, is defined as the ratio of the prescribed final *z*-displacement and *x*-displacement, in a given loading step. As a consequence, the uniaxial compression and equi-biaxial compression can be represented by BR = 0 and 1, respectively. In this study, we also considered non-equi-biaxial loading with a BR value between 0 and 1.

The critical stress at the onset of the primary bifurcation, $\sigma_{cr}$, was analytically derived as [14,16,43]

(2) $$\sigma_{cr}=\left[\frac{E_{f}}{4\left(1-\nu_{f}^{2}\right)}\right]{\left[\frac{3\left(1-\nu_{f}^{2}\right)E_{s}}{\left(1-\nu_{s}^{2}\right)E_{f}}\right]}^{2/3}$$

assuming that the stress state was uniform (in the sufficiently thin film with the cross-section area of $t_{f}\cdot w_{z}$ (or $t_{f}\cdot w_{x}$), as shown in Figure 1). It has been postulated that the critical wrinkling stress in Equation (2) is applicable to any possible biaxial wrinkling mode (including the 1D sinusoidal form and the square checkerboard) [13]. The critical strain for the primary instability modes under pure uniaxial (BR = 0) and equi-biaxial compression (BR = 1) were reported as [14,16,23,44]

(3) $${\left(e_{cr}\right)}_{1D}=\left(\frac{1}{4}\right){\left[\frac{3\left(1-\nu_{f}^{2}\right)E_{s}}{\left(1-\nu_{s}^{2}\right)E_{f}}\right]}^{2/3}$$

and

(4) $${\left(e_{cr}\right)}_{Cb}=\left[\frac{1}{4\left(1+\nu_{f}\right)}\right]{\left[\frac{3\left(1-\nu_{f}^{2}\right)E_{s}}{\left(1-\nu_{s}^{2}\right)E_{f}}\right]}^{2/3}$$

where ${\left(e_{cr}\right)}_{1D}$ and ${\left(e_{cr}\right)}_{\mathrm{Cb}}\;$are the critical wrinkling strains of sinusoidal and square checkerboard wrinkles, respectively. It should be noted that Equations (3) and (4) were apparently obtained by dividing the critical stress in Equation (2) by, respectively, the plane strain modulus and the biaxial modulus of the film layer, namely, ${\left(e_{cr}\right)}_{1D}=\sigma_{cr}/[E_{f}/(1-\nu_{f}^{2})]$ and ${\left(e_{cr}\right)}_{Cb}=\sigma_{cr}/[E_{f}/(1-\nu_{f})]$. As can be recognized from Equations (3) and (4), considering the value of *ν_f_* for the polymeric film material studied in this paper, ${\left(e_{cr}\right)}_{1D}$ > ${\left(e_{cr}\right)}_{\mathrm{Cb}}$. Moreover, for other cases of non-equi-biaxial compression, 0 < BR < 1, closed-form theoretical solutions for the critical strain do not exist; however, from our earlier numerical studies [39], the critical strain associated with any BR lies within the range between ${\left(e_{cr}\right)}_{1D}$ and ${\left(e_{cr}\right)}_{\mathrm{Cb}}$ and varies with BR monotonically, with ${\left(e_{cr}\right)}_{1D}$ being the upper limit at BR = 0, and ${\left(e_{cr}\right)}_{\mathrm{Cb}}$ being the lower limit at BR = 1. It was also demonstrated that the numerically obtained $e_{cr}$ at any BR is slightly higher than the corresponding analytical values (if available) [39]. When presenting the results in the following sections, we used the numerically obtained critical strain in the *x*-direction at the onset of bifurcation, i.e., $e_{cr}$, for the corresponding BR loading state.

## 3. Results

### 3.1. Uniaxial Compression

**Figure 2:**
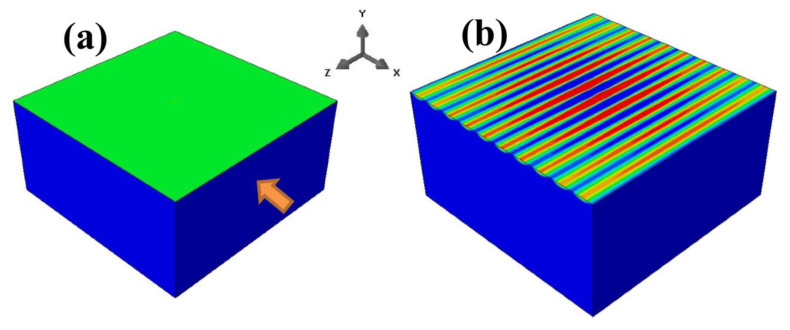

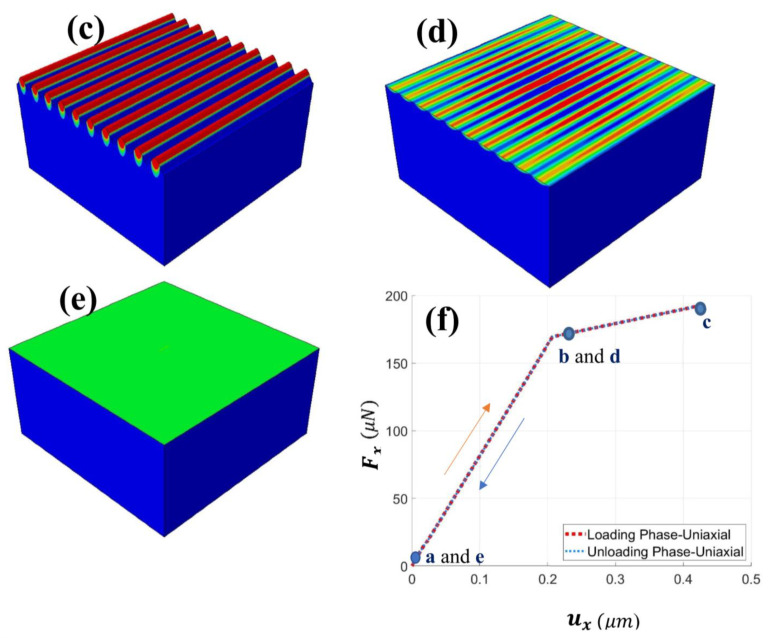
Snapshots of simulated surface patterns during the loading phase (from (**a**–**c**)) and unloading phase (from (**c**–**e**)) under uniaxial compression. (**f**) Overall load–displacement response for the entire deformation history, with the five stages (**a**–**e**) labeled along the curves.

We first considered a uniaxial compressive loading along the x-direction (BR = 0). Figure 2 shows the evolution of sinusoidal wrinkles during the loading and unloading phases. The color contours represent the extents of displacement in the out-of-plane (*y*) direction. In the loading phase (Figure 2a–c), the model was subjected to a compressive displacement from 0 to −0.42 μm (corresponding to a compressive strain of −0.0075). The extent of the applied compression in terms of normalized strain was $e_{xx}/e_{cr}≅2.0$, where $e_{xx}$ is the applied compressive strain in the *x* direction, and $e_{cr}$ is the critical wrinkling strain obtained from the simulations [36]. As can be seen, fully developed sinusoidal wrinkles were obtained at the end of the loading phase (Figure 2c). Subsequently, the analysis was continued with an unloading phase, in that the applied strain was reverted back to zero (Figure 2c–e). The wrinkles tended to disappear gradually during unloading, and a fully flat surface was in place when the compressive strain was completely removed. The overall load–displacement response resulting from the simulation is shown in Figure 2f, with the model snapshots at different stages (Figure 2a–e) labeled along the curves. It is evident that the loading and unloading responses follow the same path. At the onset of instability during loading (and at the reversal of instability during unloading), there is a distinct change in slope of the load–displacement behavior.

### 3.2. Equi-Biaxial Compression

**Figure 3:**
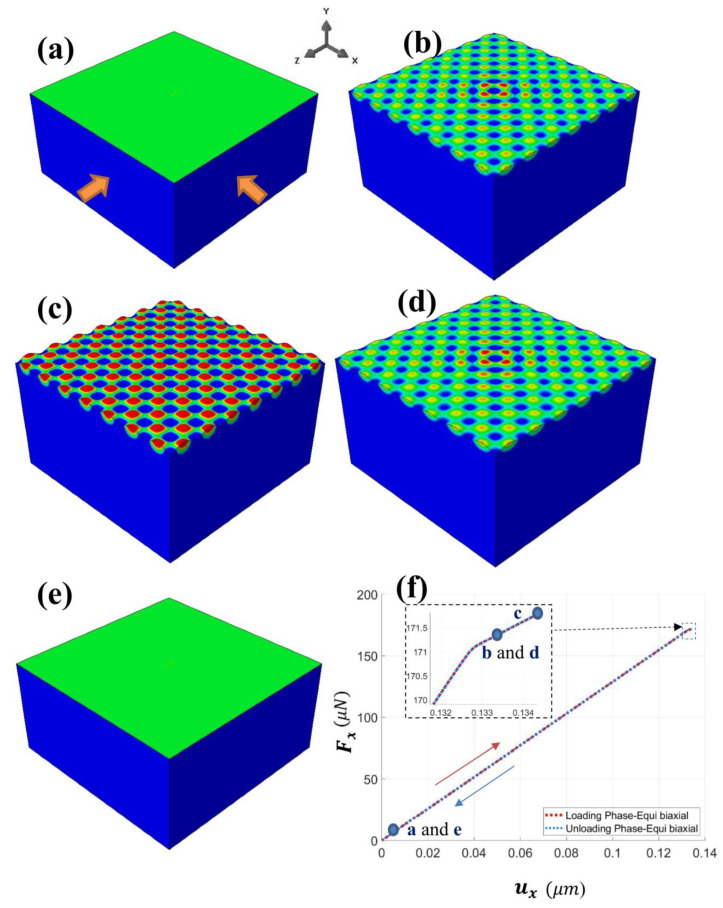
Snapshots of simulated surface pattern during the loading phase (from (**a**–**c**)) and unloading phase (from (**c**–**e**)) of the equi-biaxial compression. (**f**) The overall load–displacement response for the entire deformation history, with the five stages (**a**–**e**) labeled along the curves.

Attention was then turned to in-plane equi-biaxial loading along the *x*- and *z*-directions (BR = 1). The dominant mode of surface wrinkling at the onset of instability caused by an equi-biaxial loading is the square checkerboard pattern [15,39]. Although the pattern will eventually evolve into the labyrinth mode in the post-instability regime [39], the current section is limited to the primary mode of instability. Figure 3 shows the simulated evolution of the square-checkerboard wrinkles during loading (a–c) and unloading (c–e). The maximum equi-biaxial displacement of −0.138 μm was applied during loading, which corresponded to $e_{xx}/e_{cr}≅1.03$, with $e_{cr}$ being 0.0024. The development of a wrinkling pattern from the pre-instability flat face to the square checkerboard pattern can be clearly seen in Figure 3a–c. During the unloading phase (Figure 3c–e), the surface pattern gradually faded away, and the flat surface reappeared. Figure 3f shows the overall load–displacement response in the *x*-direction during the equi-biaxial loading/unloading. The points of the snapshots of Figure 3a–e are also labeled along the curves. As can be seen, similar to the case of uniaxial compression discussed in Section 3.1, the equi-biaxial loading and unloading responses also followed the same path. The entire history of the reversible deformation involving mechanical instabilities could thus be captured by our modeling approach in a straightforward manner.

### 3.3. Simultaneous vs. Sequential Loading

In this section, we compare the surface wrinkling patterns based on simultaneous and sequential loadings leading to the same final state of strains. Consider the case of equi-biaxial compression (BR = 1) where the target applied strain corresponds to $e_{xx}/e_{cr}≅1.03$ (maximum equi-biaxial displacement of −0.138 μm). The result of simultaneous loading is presented in Section 3.2. For a sequential loading, a uniform compression along the *x*-direction only was first applied, which was followed by a second phase of compression along the *z*-direction.

**Figure 4:**
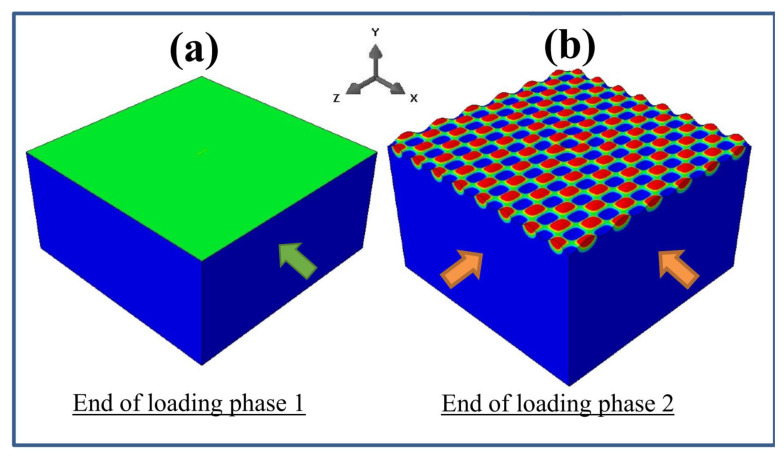

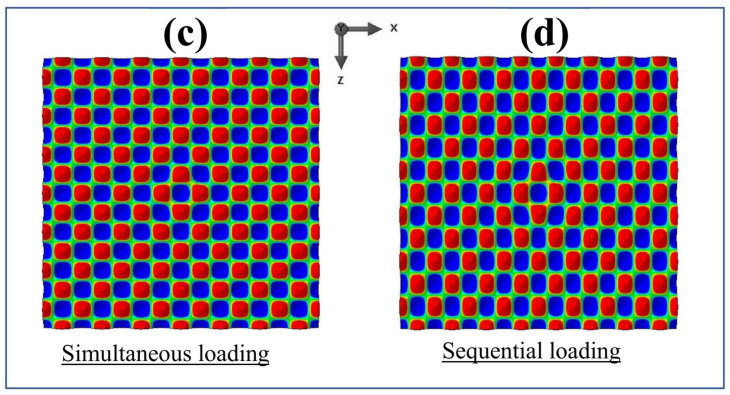
(**a**,**b**): Snapshots of simulated surface patterns during a sequential loading at (**a**) the end of phase 1 and (**b**) at the end of phase 2. (**c**) Square checkerboard wrinkling pattern obtained from a simultaneous loading. (**d**) Non-square checkerboard wrinkling pattern obtained from a sequential loading.

At the end of the second phase, $u_{x}=u_{z}=-0.138\;\mu m$, which is the same as the case presented in Section 3.2. Figure 4a,b show the surface patterns at the end of each phase of the sequential loading. After phase 1, the surface remained flat because the applied displacement had not reached the critical value for uniaxial compression (see Figure 2f). At the end of phase 2, a checkerboard-like pattern appeared, but it was not of the exact square type, as can be seen from the different numbers of waves along the *x*- and z-directions.

Figure 4c,d show the top views of the surface patterns resulting from the simultaneous and sequential loadings, respectively, for a direct comparison. Despite the same end state of equi-biaxial compressive strains, the sequential loading led to a non-square checkerboard pattern, as opposed to the perfect square checkerboard pattern obtained with the simultaneous loading. It is apparent that the mechanical instability rendered the simple elastic deformation history-dependent. Such a dependence may be attributed to the change in the domain geometry after the first phase of compression along the *x*-direction. The longer span in the *z*-direction at the end of the first phase (or equivalently, at the start of the second phase), coupled with the fixed constraint maintained in the *x*-direction during the second phase, contributed to the formation of a rectangular type of checkerboard pattern. The present finding also brings about the possibilities for creating variants of wrinkle patterns controlled by the deformation path, which is worthy of further theoretical and experimental investigations.

### 3.4. Simultaneous vs. Sequential Loading: Further Instability Modes

**Figure 5:**
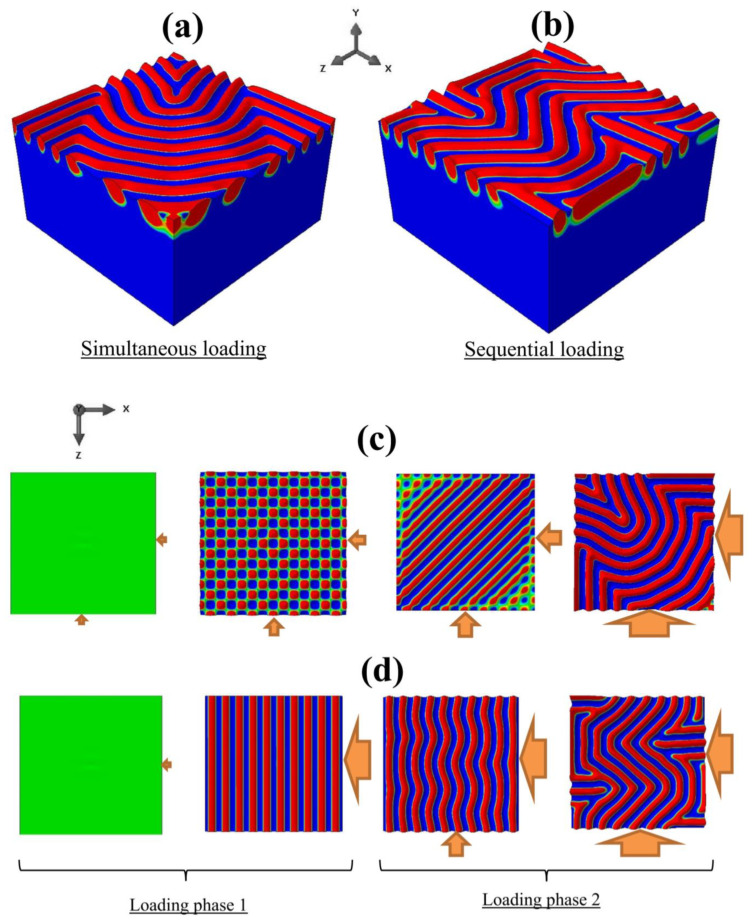
Snapshots of the simulated wrinkling patterns for BR = 1.0 and $e_{xx}/e_{cr}≅3.80$, for the cases of (**a**,**c**): simultaneous loading and (**b**,**d**): sequential loading. The wrinkle configurations at the end of each simulation are presented in (**a**,**b**). The evolution of the wrinkling patterns (starting from a flat surface until reaching the target strains) are shown in (**c**,**d**). The directions of the applied displacements are also schematically shown.

The history dependency of the surface wrinkling patterns was not limited to the primary bifurcation mode. It was even more apparent as the deformation extended into the post-instability regime. For demonstration, consider the same problem in Figure 4, but the specimen was now subjected to a higher maximum equi-biaxial target displacement of −0.51 μm (which corresponds to $e_{xx}/e_{cr}≅3.80$). Figure 5a,b show the final surface states for the simultaneous and sequential loadings, respectively, with very different wrinkle patterns. Their temporal developments for the cases of simultaneous and sequential loading are presented in Figure 5c,d, respectively. Under simultaneous loading (Figure 5c), the primary instability mode was the square checkerboard, which, with further straining, eventually evolved into a labyrinth pattern. Under sequential loading (Figure 5d), the primary instability was the 1D mode, since phase 1 involved only a uniaxial compression applied in the *x*-direction. During phase 2, the superposition of *z*-compression led to a herringbone-like structure, which eventually became a labyrinth. It was again observed that the same final strain states in Figure 5c,d via different deformation histories, had distinctly different surface patterns.

## 4. Discussion

The results presented in Section 3.3 and Section 3.4 clearly demonstrated the path dependence of surface instability, even when the materials are treated as linearly elastic. It is worthy of note that except for very rare cases [45], a true square-checkerboard wrinkling configuration has never been obtained in real experimentation [15]. Various potential reasons, such as a curved initial geometry and a special constitutive behavior of the materials, have been proposed [15,25,30,39,46]. The finding presented in Figure 4 of this work, under equi-biaxial compression, points to yet another possibility, i.e., that deviations from the true simultaneous uniform loading along the two directions (e.g., slight interruptions of loading continuity in any one direction) may create a favorable condition for the non-square-type checkerboard pattern.

**Figure 6:**
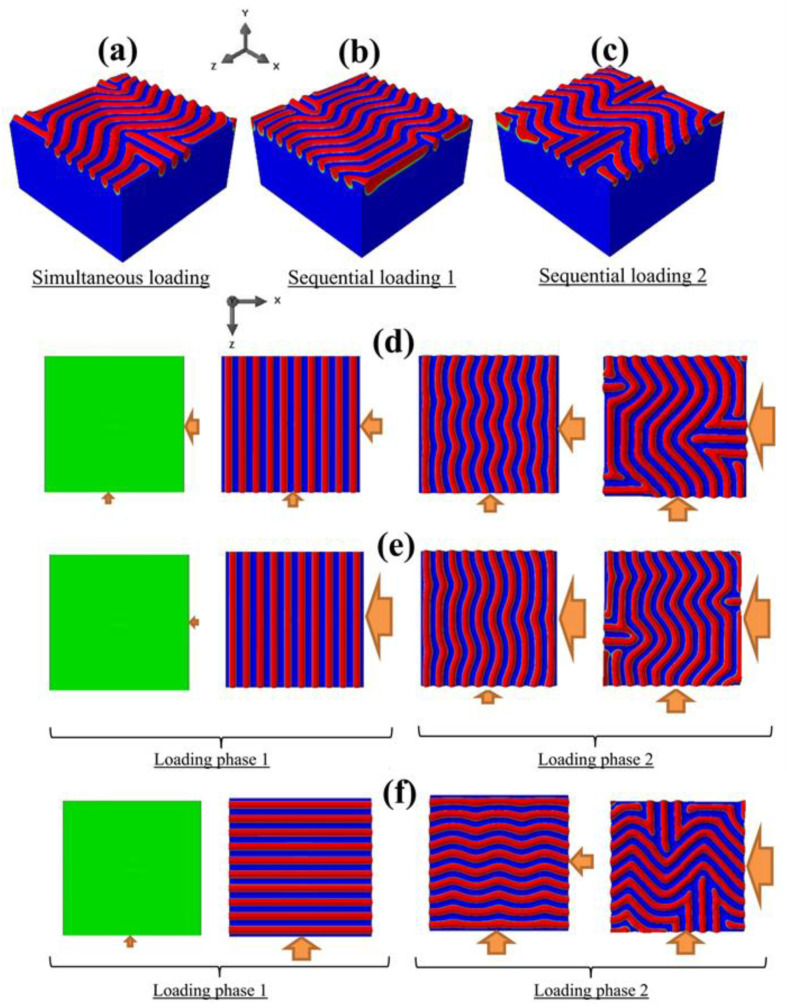
Snapshots of the simulated wrinkling patterns for BR = 0.7 and $e_{xx}/e_{cr}≅3.50$, for the case of (**a**,**d**): simultaneous loading, (**b**,**e**): sequential loading 1, and (**c**,**f**): sequential loading 2. The wrinkling configurations at the end of each simulation are presented in (**a**–**c**). The evolution of the wrinkling patterns (starting from a flat surface until reaching the target strains) are shown in (**d**–**f**). The directions of the applied displacements are also schematically shown.

Aside from the uniaxial and equi-biaxial loadings considered above, one can explore a non-equi-biaxial loading with the BR value falling between 0 and 1. Here, we will discuss the case of BR = 0.7. Three sets of simulations with the maximum applied displacements of $u_{x}=-0.51\;\mu m$ and $u_{z}=-0.357\;\mu m$, corresponding to a final deformation of $e_{xx}/e_{cr}≅3.50$ ($e_{cr}=0.0026$), were first considered: (i) simultaneous loading along the *x*- and *z*-directions, (ii) two-phase sequential loading with *x*-compression applied first and *z*-compression applied afterward, (iii) two-phase sequential loading with *z*-compression applied first and *x*-compression afterward. In the following, the two sequential cases (ii) and (iii) are also referred to as “sequential loading 1” and “sequential loading 2,” respectively. The numerical results are shown in Figure 6, with Figure 6a–c displaying the final wrinkle patterns at the end of simultaneous loading, sequential loading 1, and sequential loading 2, respectively. As can be seen, different wrinkle patterns were obtained with different deformation paths, while the loading biaxiality was identical (BR = 0.7) at the end of each simulation.

The temporal evolutions of instability are presented in Figure 6d for simultaneous loading, Figure 6e for sequential loading 1, and Figure 6f for sequential loading 2, all from a flat pre-instability state well into post-instability. Under simultaneous loading at BR = 0.7 (Figure 6d), the primary 1D instability mode transformed into a herringbone and later a labyrinth pattern, which is consistent with our previous studies and other experimental/analytical investigations reported in the literature [15,25,39,44,47]. A qualitatively similar evolution path was observed for the case of sequential loading 1 (Figure 6e), where the *x*-compression was applied first (during loading phase 1). It should be noted that, although the evolution paths in Figure 6d,e are similar, the actual herringbone and labyrinth surface patterns were different. This difference can be attributed to the more prominent sinusoidal wave amplitude in the case of sequential loading 1 at the end of loading phase 1, since the maximum applied displacement of $u_{x}=-0.51\;\mu m$ was already reached, while in the case of simultaneous loading, the mode transformation from a 1D to a herringbone pattern happened much earlier. Sequential loading 2, where the *z*-compression was applied first, triggered the formation of 1D wrinkles in the perpendicular direction (Figure 6f). Note that, with BR = 0.7, the applied displacement at the end of the loading phase 1 was $u_{z}=-0.357\;\mu m$, which rendered yet another sinusoidal wave amplitude. A subsequent *x*-compression during the loading phase 2 therefore led to different forms of herringbone and then labyrinth configurations. It is worth noting that the wrinkle amplitude was directly proportional to the intensity of the applied strain, as documented in many analytical/experimental/numerical studies in the literature [14,15,39].

**Figure 7:**
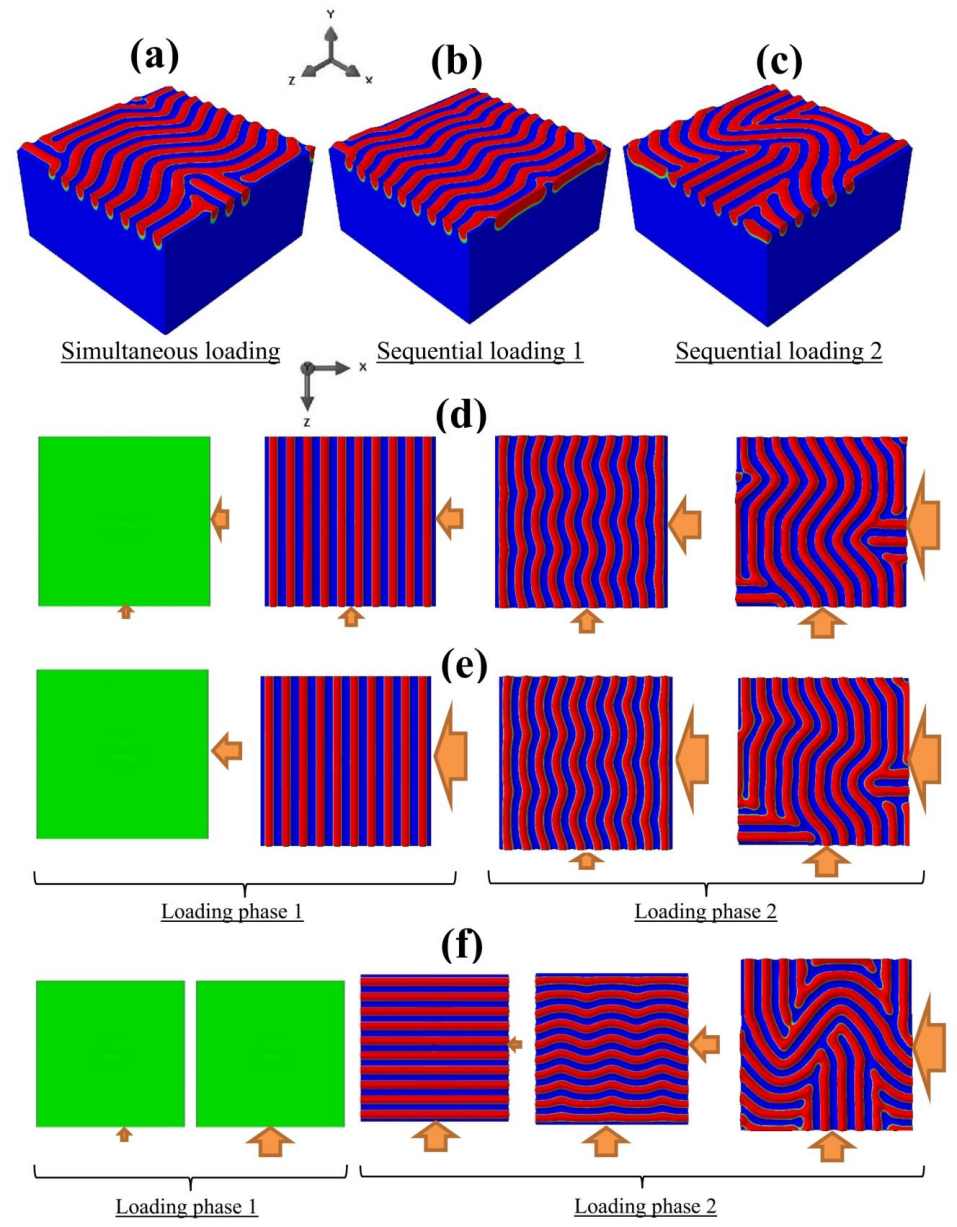
Snapshots of the simulated wrinkling patterns for BR = 0.7 and $e_{xx}/e_{cr}≅1.96$, for the case of (**a**,**d**): simultaneous loading, (**b**,**e**): sequential loading 1, and (**c**,**f**): sequential loading 2. The wrinkling configurations at the end of each simulation are presented in (**a**–**c**). The evolution of the wrinkling patterns (starting from a flat surface until reaching the target strains) are shown in (**d**–**f**). The directions of the applied displacements are also schematically shown.

Figure 7 shows the results for another set of simulations at BR = 0.7, with a smaller target strain of $e_{xx}/e_{cr}≅1.96$. The maximum applied displacements were $u_{x}=-0.284\;\mu m$ and $u_{z}=-0.199\;\mu m$. The final wrinkling configurations are shown in Figure 7a–c, respectively, for the three cases of simultaneous loading, sequential loading 1 (*x*-compression first), and sequential loading 2 (*z*-compression first). The history-dependent wrinkle patterns are again evident. The deformation progression is shown in Figure 7d–f for the cases of simultaneous loading, sequential loading 1, and sequential loading 2, respectively. As in the case of Figure 6, simultaneous loading in Figure 7d and sequential loading 1 (*x*-compression first) in Figure 7e resulted in a similar kind of pattern evolution, but the detailed wrinkle morphologies were not the same. An interesting pattern evolution, however, was observed for sequential loading 2 in Figure 7f (with *z*-compression first). Here, the primary instability did not occur at the end of the loading phase 1. This was due to the smaller target strain; the maximum applied displacement ($u_{z}=-0.199\;\mu m$) corresponded to the applied strain of $e_{zz}=-0.0036,$ which was still below the critical strain of $e_{cr}≅-0.0037$. At the beginning of the second loading phase, only a slight compression of *u_x_* triggers the formation of 1D wrinkles in the original *z*-direction. With increasing *x*-compression, the bifurcation mode changed to herringbone and then labyrinth.

Figure 5, Figure 6 and Figure 7 demonstrated that the history dependency is not limited to the primary form of instability. The development of further wrinkle patterns can be influenced by the sequence of loading and its extent. The findings in this work also suggest wide-ranging possibilities for generating variants of wrinkle patterns controlled by the deformation history for various functionalities, including optics [6], friction [46], wetting [5], antifouling [48], etc. It is also worthy of note that the embedded imperfection technique used here is particularly suited for multi-phase loading processes such as cyclic deformation. The need for re-defining imperfections in each loading phase can be completely avoided. Future studies may incorporate inelastic and/or damage constitutive behaviors to simulate complex cyclic deformation histories and fatigue damage of film–substrate systems.

## 5. Conclusions

This paper presents a numerical study on the formation of wrinkle patterns in thin film/compliant substrate material systems. The embedded imperfection approach was successfully applied for direct three-dimensional finite element simulations of surface wrinkling, accounting for loading/unloading and the effect of the deformation history. The modeling technique is robust and easy to implement and avoids the need of re-defining imperfections in each loading phase. Other salient findings and concluding remarks are summarized below.

- Within the linear elastic framework free of material damage, deformation instabilities in the form of surface wrinkles are recoverable under uniaxial and equi-biaxial loading and unloading.
- Using different deformation paths to reach the same equi-biaxial and non-equi-biaxial states, however, results in different wrinkle configurations.
- The history dependency is applicable to the primary instability mode as well as to subsequent transformations of wrinkle patterns.
- The history dependency also raises the possibilities of devising special loading sequences in actual experiments to achieve specific surface patterns.
- This study paves the way for future explorations involving complex deformation paths, inelastic deformation and damage, and cyclic responses.

## Data Availability

Not applicable.
